# Facilitators and Barriers to Being Physically Active in a Rural Hawai‘i Community: A Photovoice Perspective

**DOI:** 10.31372/20180304.1015

**Published:** 2018

**Authors:** Siosaia F. Hafoka, Sara J. Carr

**Affiliations:** a University of Hawai‘i at Māanoa, Honolulu, HI, USA; b Northeastern University, Boston, MA, USA

**Keywords:** Native Hawaiian, Pacific Islanders, Photovoice, physical activity, community based participatory research, rural health, active living, health disparities

## Abstract

A large body of existing literature suggests associations between perceptions of the environment and physical activity status, but very few studies have examined perceptions among Native Hawaiian and Pacific Islander (NHPI) communities. The purpose of this study was to examine perceptions of the active living environment in Hawai‘i in rural communities with a high proportion of NHPI. A total of thirteen adults were purposefully selected to participate in the study, and the Photovoice method was used to capture their perceptions. Three sessions were used to describe the purpose of the study, select and describe photographs, and identify emerging themes. A total of nine overarching themes were identified by participants and placed into three categories. Participants identified facilitators to being physically active, but also identified several barriers in their community such as the lack of available physical and built amenities, social norms, and safety. Participants proposed four action items to improve their active living environment: promote programs that are organized by community members, contact the local university to provide access to physical activity equipment, contact Honolulu City & County officials to voice concerns regarding barriers that prevent physical activity in the community, and working with employers in the community to create policies that promote physical activity at the workplace.

## Introduction

The benefits of physical activity have been well documented (Warburton, Nicol, & Bredin, 2006). Several studies have shown that physical and mental health benefits are extended to all age groups who are physically active (Poitras et al., 2017; Turner, Lira, & Brum, 2017). Existing literature report that perceptions of the environment can influence physical activity (Lamit, Majid, Shafaghat, & Keyvanfar, 2012; McGinn, Evenson, Herring, Huston, & Rodriguez, 2007; Troped, Tamura, Whitcomb, & Laden, 2011). Previous studies have used qualitative research methods in different settings to understand barriers and facilitators to being physically active, which may not have been identified if the studies solely relied on quantitative methods (Cleland et al., 2015; Ferrer, Ruiz, & Mars, 2015; Hume, Salmon, & Ball, 2014; Rader et al., 2015).

The Native Hawaiian and other Pacific Islander (NHPI) population is one of the fastest growing racial groups in the United States (Albright et al., 2017). Studies using community based participatory research methods among NHPI are very limited, and even fewer studies examine their perception of the environment in rural areas (Ro & Yee, 2010). The purpose of this study was to answer an important question that could be used to increase opportunities for physical activity: what do Native Hawaiian and other Pacific Islanders (NHPI) perceive as barriers and facilitators to being physically active in their rural community? This qualitative study employs the Photovoice method, “a participatory means of sharing expertise and knowledge” through photographs captured by study participants (Wang & Burris, 1997).

## Method

Kahuku, La‘ie, and Hau‘ula are rural towns located on the north and northeast shores of the island of O‘ahu. They were selected for the study because all three communities have a proportion of NHPI residents that are higher than that of the state’s (Table 1). According to the Census (2015), La‘ie is the most populated of the three towns (6,419) and Kahuku is the smallest by population (3,292). Median incomes in Kahuku and Hau‘ula are both below the state’s median income ($61,250 and $65,625, respectively). The median income for La‘ie residents is well over the state’s median income, at $86,731, and they also have the lowest proportion of NHPI residents.

**Table 1:** Observed Community

	Kahuku	La’ie	Hau’ula	Hawai’i
Population, *N*	3,292	6,419	5,555	1,360,301
Native Hawaiian or other Pacific Islander, % (*n*)	59.5 (1,960)	56.8 (3,292)	70.2 (3,904)	25.7 (350,288)
High school graduate or higher, % (*n*)	87.4 (2,285)	97.9 (6,009)	87.4 (3,625)	90.7 (1,233,793)
Median household income, dollars	61,250	86,731	65,625	68,201
Persons below poverty level, % (*n*)	14.9 (490)	13.2 (847)	12.9 (716)	11.2 (152,353)

*Source:* Census 2015 (www.factfinder.census.gov).

### Participants

For this study, fifteen community members were purposefully selected from a larger study of over 300 participants. As a resident of the observed community, the primary author selected a convenient sample from all three towns based on a larger study of over 300 participants. Two participants were unable to attend any of the scheduled meetings, which left a total of thirteen community members (five males, eight females). Each town was represented among the participants, and the mean age was 32 years (range 29–49 years).

### Study Design

Approval from the University of Hawai‘i’s Institutional Review Board was received prior to initiating the study.

Participants were asked to attend three sessions between April and May 2017 to complete the project. As an incentive for attendance, dinner was provided at each session. Eleven participants attended all three sessions. Two participants were unable to attend Session Three, so they met at a different scheduled time. All three sessions were held in a private room at the Brigham Young University-Hawai‘i campus and were less than 90 min each.

### Session One

The consent form was reviewed at the beginning of Session One. The consent form explained that the risks to participating in this study were very minimal—participants were not asked to modify or change their daily routines. Participants were also informed of possible benefits that may result from this study—information that could be used to inform elected officials and community members of facilitators and barriers to being physically active in the community. All the participants signed the consent form before continuing with the first session.

The purpose of the study and the Photovoice method was explained in Session One. Although digital cameras were offered for the study, participants requested that they use their personal cell-phone to capture photographs, which has also been used in previous Photovoice studies (Alcazar, Raber, Lopez, Markham, & Sharma, 2017; Williams et al., 2013). All cell-phones were checked to see if they had camera capabilities, text messaging, and email services. Participants were not allowed to photograph images, portraits, or people who could be easily identified. Any photographs with identifiable faces or body features (piercing or tattoos) would not be accepted and would be deleted immediately by the primary author (SH).

Participants were instructed to take photographs that served as a barrier or facilitator to them being physically active. A “barrier” was described as something (physical, social, economic, or perceived) that prevented them from being physically active. A “facilitator” was described as something (physical, social, economic, or perceived) that allowed them to be physically active. “Physical activity” was defined as any movement that was done for leisure or transportation (Mahmood et al., 2012). They were instructed to take pictures of barriers or facilitators they would encounter on a normal day. Participants were provided with examples from a Photovoice assignment that SH completed for a qualitative methods course as well as examples from similar Photovoice studies. There were no minimum requirements or upper limit to the number of photographs they could take. Participants were given one week to take photographs (Cahill & Suarez-Balcazar, 2012; D’Alonzo & Sharma, 2010; Mmari et al., 2014). Photographs were emailed or texted to SH throughout the week, who compiled the photographs taken by each participant.

### Session Two

All the photographs were printed by SH at the end of the one-week period and then distributed to each participant at the start of Session Two. The purpose of Session Two was to discuss their photographs and generate themes. Participants were asked to select up to three of their photographs that could best describe barriers and facilitators to physical activity. Before beginning the discussion, participants gave consent to be audio-recorded. To initiate the discussion, the mnemonic questions, SHOWeD, were written on a whiteboard:

S: What do you See here?

H: What is really Happening here?

O: How does this relate to Our lives?

W: Why does this situation, concern, or strength exist? and,

D: What can we Do to improve the situation, or to enhance these strengths?

*(**Wang, Morrel-Samuels, Hutchison, Bell, & Pestronk, 2004**)*

Each participant began by answering the SHOWeD questions. After each participant’s description of their photograph, the entire group was encouraged to share their thoughts on the image. The group was encouraged to comment or ask any follow-up questions to the individual describing their photograph. Responses from the “D” in SHOWeD were discussed and all thirteen participants felt a need to create specific action items in response to their photographs and discussion. The group decided to move on to the next photograph if they felt the answers to the SHOWeD questions and discussion sufficiently described what the participant intended to describe. The audio recording from Session Two was transcribed verbatim and participants were identified alphanumerically. The audio file and transcript were securely stored on a flash drive that was only accessible by SH.

### Session Three

Session Three was used to review the audio transcription with participants, and collaboratively identify themes and categories from Session Two. Consistent with the Photovoice procedures, all themes were participant-driven rather than investigator-driven (Hennessy et al., 2010).

### Data Analysis

Field notes were recorded by SH throughout (before, during, and immediately after) the three Photovoice sessions to provide context and inform the analysis of the discussions (Carlson, Engebretson, & Chamberlain, 2006; Phillippi & Lauderdale, 2017). The notes were used to capture any side comments and non-verbal cues (facial expressions and body language) of the participants. The thematic analysis was a multi-step and collaborative process (Braun & Clark, 2006; Findholt, Michael, Davis, & Brogoitti, 2010). First, the audio recording from Session Two was transcribed verbatim and then distributed to participants during Session Three. Photographs were paired with their description in the transcript, and then categorized as barriers, facilitators, or barriers and facilitators. Repeated phrases (e.g. “it’s not safe to walk around…” and “I don’t think its safe…”) were automatically grouped into a common theme and category (Ryan & Bernard, 2003). Comments that were not repeated in the transcript but were related to an existing theme were also grouped together. Any disagreement among participants regarding specific themes were discussed, and any changes to the themes were made when a consensus was achieved (Belon, Nieuwendyk, Vallianatos, & Nykiforuk, 2016).

## Results

A total of 81 photographs were taken and submitted (range 1 to 23 per person). Of that total, 27 photographs (range 1 to 3 per person) were selected for discussion. Nine overarching themes were identified, and photographs were divided into three categories: barriers, facilitators, and themes that were perceived as both barriers and facilitators.

### Barriers to Physical Activity

A total of five major themes (perceived safety, availability of built amenities, accessibility to existing built amenities, social norms, and the weather) emerged that were related to barriers to physical activity. The most common theme among barriers was the perceived safety among participants.

One participant expressed safety concerns about the existing bike path between Kahuku and La‘ie, saying:

“The issue was the lack of a guardrail. There’s not a guardrail that runs along that would protect the bike path. I’ve seen a lot of cars serve off to the side. So, I thought that was a huge barrier for me running on the bike path.” (Participant A, Male, 31)

**Figure 1:**
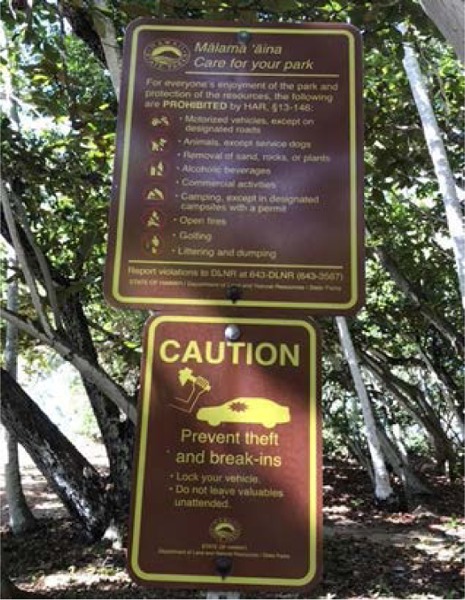
Beach theft safety sign.

Another participant photographed signs at a beach parking lot that also echoed concerns for safety (Figure 1):

“The first sign is just the rules of the park and the second one is 'Prevent theft and breakins’. Especially over there, I never leave my car because I feel it can get broken into. That’s what I feel like, just the thought of leaving my car that far away.” (Participant B, Male, 37)

**Figure 2:**
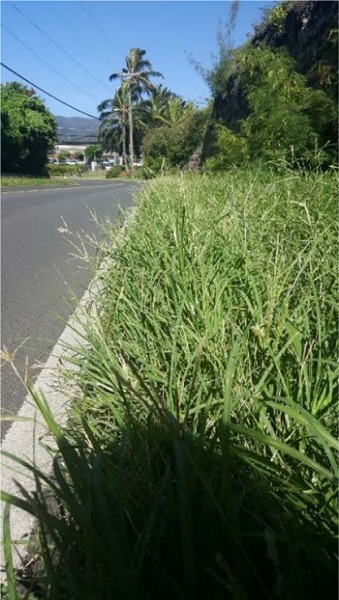
Weeds along street shoulder.

The availability and accessibility of physical amenities was a common theme from the group discussion. Another participant noted (Figure 2):

“There’s no sidewalk on the main road up at the Point. We’re constantly fighting traffic, and the grass that we’re supposed to be walking on, to say off the road, isn’t always mowed. Like right now, it’s up to your knees so then it forces you to walk on the road and causes a safety hazard.” (Participant C, Female, 34)

### Facilitators to Physical Activity

**Figure 3:**
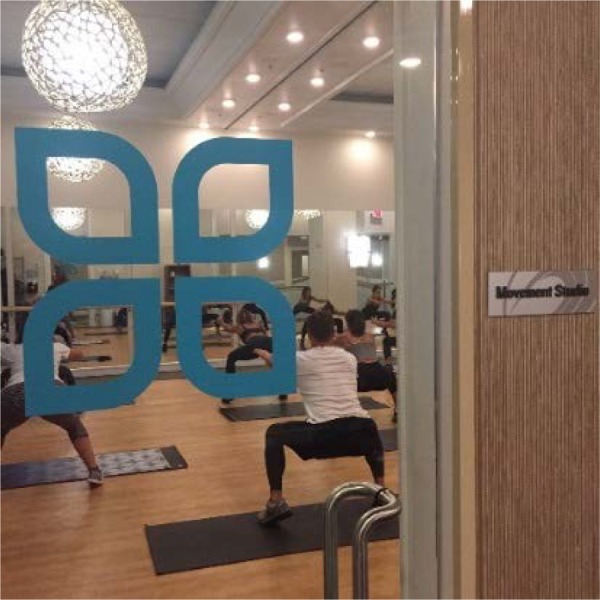
Turtle Bay fitness center.

Major themes that emerged as facilitators to physical activity were: availability of outdoor amenities, accessibility to outdoor amenities, work duties, and cultural activities. There were also recreational facilities in the area that participants identified as facilitators to being physically active (Figure 3).

“I took a picture of the fitness room at Turtle Bay. It’s a place that I work out at and they have a lot of organized classes, which is why I really like it.” (Participant D, Female, 34)

**Figure 4:**
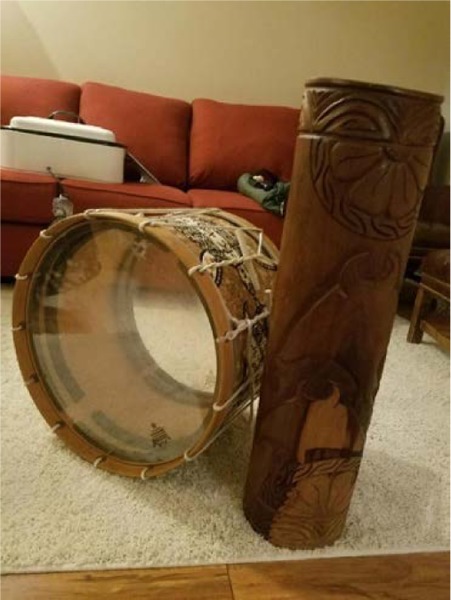
Cultural drums.

The fitness center at the Turtle Bay resort in Kahuku is open from 6 am–10 pm and requires a 24-h fitness card to access it after regular hours. Free weights, stationary bicycles, and machines are also available in the fitness room. The Polynesian Cultural Center (PCC), one of the largest employers in the area provides opportunities for employees to be physically active by performing cultural dances and activities. One participant photographed drums that are used for work (at PCC) and cultural performances in the community, resulting in another emergent theme, specific to the NHPI community of cultural activities and norms (Figure 4).

“I have drums – Tahitian and Samoan drums – that I can use to stay physically active. I practice in a group or by myself.” (Participant E, Male, 42)

### Barriers and Facilitators Overlap

**Figure 5:**
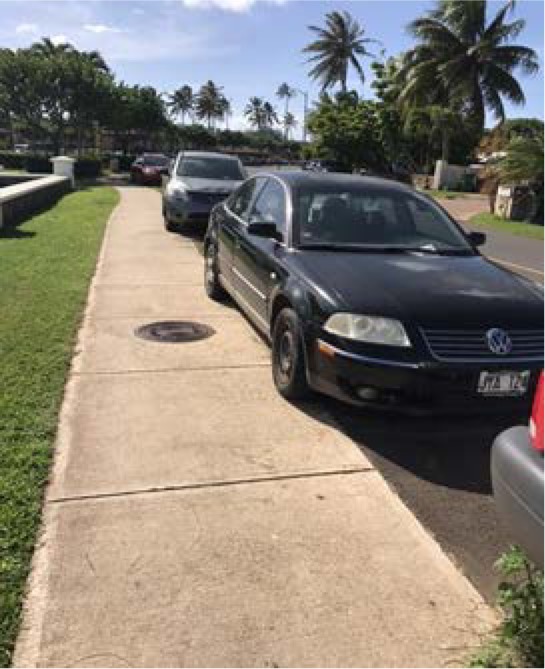
Vehicle parked on sidewalk.

The participants’ perceptions of barriers and facilitators are unique in that they are community-specific, such as the use of cultural drums, and the community’s acceptance to using their vehicle when traveling even short distances. At times, what may be viewed as a facilitator to being physically active could also be viewed as a barrier (Walia & Liepert, 2012). For example, the bike path was observed as a facilitator that participants could use to be physically active, but it was also seen as a barrier because of a perceived safety issue—no guardrail to serve as a physical barrier between bike path and Kamehameha Highway. Sidewalks were recognized by participants as a facilitator to being physically active; however, it is common to find vehicles parked on the sidewalk, making it dangerous for pedestrians (Figure 5).

“I hope this isn’t somebody’s car, but they’re parking on the sidewalk. So, there’s this sidewalk, but for whatever reason, he’s parking on the sidewalk. So, when you’re walking with a stroller and you have to wait, stop, and go around the grass. They did the right thing by making the sidewalk, but I can’t even fully use the sidewalk.” (Participant B, Male, 37)

### Action Items

The “D” in SHOWeD allowed participants to discuss how they could improve the situation or enhance the strength that was captured in the photograph. Four participant-selected action items were created as a result of the Photovoice sessions:

1. Identify and disseminate information on existing programs within the community that provide opportunities for physical activity. These programs include a yoga class on the beach, a kickboxing and Jiu Jitsu class, a “Walk with a Doc” program that uses the Malaekahana Bike Path, and free Zumba classes held at churches in the community.
  “In the BYU parking lot there are always groups of people walking in the parking lot because that’s the only space to be able to run or whatever. In the past we’ve gone there to lift weights. There are groups that have been created that use resources that we do have. There have a kickboxing class at [John’s] house. And a Jiu Jitsu class, and yoga on the beach.” (Participant C, Female, 34)
2. Contact BYU-H representatives to add outdoor exercise equipment across campus. Some participants recalled a time when exercise equipment was placed at several different locations across BYU-H’s campus. Those participants would like to see if BYU-H can bring something similar back to campus for community members.
  “Remember when BYU-H used to have equipment like Kapiolani Park? They probably had 20 stations all around campus. Pull up bar and all kinds of things. And I don’t know why they took it out. We were little kids. It would be nice if BYU-H could put those back in.” (Participant B, Male, 37)
3. Contact representatives at Honolulu City & County and the State Department of Transportation offices. Participants expressed interest in contacting local elected officials to voice their concerns to address some of the barriers they identified in this project, such as cars parking in bike lanes and sidewalks, creating more sidewalks and crosswalks, creating a physical barrier between Kamehameha Highway and the Malaekahana Bike Path, and making improvements to local parks (like adding lights to use at night) and high school (improving current track conditions).
  “I think promoting awareness and communicating with the City and County, so we would be able to have sidewalks to walk safely and for the City and County if they could put the street lines up so everybody knows the streets and shoulders.” (Participant F, Female, 29)
  “People in our community are still creative in how they are able to be physically active.” (Participant C, Female, 34)
4. Create workplace policies that would promote physical activity. Although the communities’ largest employers (BYU-H, the Polynesian Cultural Center, and Department of Education schools) can be accessed by using public transportation, walking, or bicycling, most employees drive to work.

Worksite programs and incentives could be offered for employees. Some participants expressed concerns about showing up to work sweaty if they decided to ride the bike or walk that morning.

“For me, I walk to work but I don’t like to do it. It’s not that I’m lazy, it’s that I don’t want to be sweaty all day at work. I’m going to be there 8 hours and I don’t want to be sweaty and smell. It’s not necessarily being lazy at times, part of it is more hygiene.” (Participant D, Female, 34)

Showers could also be something that worksites could look at if hygiene is something that would prevent employees from being physically active.

Participants suggested that employers reserve escalators strictly for people who are disabled.

“What if, at work and other public places, elevators were only allowed for people who were disabled and everybody else had to walk upstairs? Something to promote physical activity at work.” Participant G, Female, 36)

## Discussion

The purpose of this study was to engage NHPIs in identifying barriers and facilitators to physical activity in their community. To the knowledge of the authors, this study is the first to examine barriers and facilitators to physical activity through the lens of NHPI communities by using Photovoice method. The results provide insight of what NHPIs perceived as barriers and facilitators to being physically active in their rural community. Findings can be used to examine potential environmental changes to improve security, access, and availability for physical activity opportunities. Although participants and pictures were from different towns, the themes that emerged from their photographs were similar.

Some of the barriers that participants identified were similar to barriers found in previous studies examining non-urban areas such as feeling unsafe, the lack of recreational facilities, sidewalks, and bicycling facilities (Findholt et al., 2010; Seguin, Connor, Nelson, LaCroix, & Eldridge, 2014; Walia & Liepert, 2012; Yousefian, Ziller, Swartz, & Hartley, 2009). Of the three towns observed, La‘ie had the highest median income ($86,731), and some of the facilitators which were identified in La‘ie are consistent with studies where areas with higher income levels have, such as sidewalks and safer active transportation environment around schools (Gibbs, Slater, Nicholson, Barker, & Chaloupka, 2012).

One limitation to this study was that there were only thirteen participants. Although a review of 37 Photovoice studies by Catalini and Minkler (2010) found a median of thirteen participants, the limited sample size suggests that all the community’s perceived barriers and facilitators to physical activity may not have been captured. The results may not be generalizable to other NHPI populations, as the participants in this study were all married—non-married adults may perceive differences in barriers and facilitators to being physically active in their community. Perceptions may also differ among older (>50 years) or younger (<18 years) populations, as participants of this study were between 29 and 49 years old. Even with the limited sample size, similar photograph selections and agreement during discussions suggest that participants were able to portray a comprehensive view on what they perceived as barriers and facilitators to physical activity.

The most notable strength to this study was that community perceptions were obtained using a qualitative method. Unlike quantitative methods such as a questionnaire, the participant’s responses may not illustrate the real magnitude or meaning of their perception. The discussions and descriptions of the photographs provided valuable insight to the barriers and facilitators perceived by the community, which may not have been achieved if a quantitative method was used. All barriers, facilitators, and emerging themes were identified by the participants instead of the researcher.

Interestingly, all three towns are located along the north and northeast shores of the island, giving residents access to public beaches in the area. However, very little mention was made by the participants of the beach being used for physical activity.

Participants discussed indoor facilities at BYU-H and how students and faculty can use it. The university has an indoor gym, indoor basketball courts, outdoor tennis courts, and an outdoor swimming pool that could greatly benefit the community. It is not open to the public, however, there may be opportunities to discuss possible agreements with the university to use their recreational facilities. BYU-H recently eliminated all collegiate sports programs but have still maintained their sports facilities. A “joint use agreement” (JUA) can be a formal or informal agreement between a school and the community to use facilities conducive to physical activity (Young et al., 2014) and is an objective under the Healthy People 2020 Plan, reading:

PA-10: Increase the proportion of the Nation’s public and private schools that provide access to their physical activity spaces and facilities for all persons outside of normal school hours (that is, before and after the school day, on weekends, and during summer and other vacations).

A study by Maddock, Choy, Nett, McGurk, and Tamashiro (2008) on O‘ahu examined the implementation of a JUA with high school facilities located in a low-income and high immigrant area of Honolulu. The JUA increased opportunities for physical activity, but also provided other benefits such as making new friends, kept youth out of trouble, and promoted healthy lifestyles (Maddock et al., 2008). If similar benefits could be replicated in the observed communities, a JUA with BYU-H should be a priority among community members.

The discussion proved to be insightful, as participants explained that although they may have focused more on barriers in their community, there are still several opportunities to be physically active in the community (e.g., walking to the park, lift weights at a neighbor’s house, walking or bicycling to work, cultural practices). Based on the findings from this study, future research should examine how this community views the beach and beach activities as a form of physical activity. Since BYU-H and public grade schools are among the largest employers in the area, it would also be worth exploring the feasibility of JUAs with these institutions.

## Acknowledgments

The authors would like to thank the community members who participated in this study and for your perspective on environmental factors that influence physical activity.

## Declaration of Conflicting Interests

The authors have no financial interest or potentially conflicting interests that could have any influence on the study.
